# Thymoma and Myasthenia Gravis: An Examination of a Paraneoplastic Manifestation

**DOI:** 10.7759/cureus.34828

**Published:** 2023-02-10

**Authors:** Mira Itani, Yarden Goldman Gollan, Kristin Ezell, Mohamed Mohanna, Saad Sabbagh, Caoimhin Mears, Katrina A Mears, Barbara Dominguez, Doron Feinsilber, Zeina Nahleh

**Affiliations:** 1 Hematology-Oncology, Cleveland Clinic Foundation, Weston, USA; 2 Hemaology-Oncology, Ross University School of Medicine, Weston, USA; 3 Hematology-Oncology, Ross University School of Medicine, Weston, USA; 4 Hematology-Oncology, Cleveland Clinic Florida, Weston, USA; 5 Ophthalmology, Retina Consultants of Southwest Florida, Fort Myers, USA

**Keywords:** thoracic surgery, oncology, neurology, case report, paraneoplastic syndrome, myasthenia gravis (mg), thymoma

## Abstract

Thymoma is a rare type of malignancy but is considered one of the most common neoplasms that occur in the anterior mediastinum. A large proportion of thymomas are associated with paraneoplastic syndromes, such as myasthenia gravis. Whenever feasible, the standard of care for the treatment of thymoma should focus on the control of paraneoplastic syndromes, surgical resection, and adjuvant therapy if appropriate.

A 36-year-old female patient with a significant past medical history of obesity and iron deficiency anemia who underwenten bloc resection of thymoma three months prior now presented to the benign hematology clinic to establish care for the management of anemia. Upon review of systems, the patient incidentally reported fatigue, weakness with repetitive motion, occasional blurred vision, headaches, and exertional dyspnea. Physical examination was positive for horizontal nystagmus. Given the patient's history and clinical findings, suspicion of myasthenia gravis was high. Further work-up demonstrated anti-acetylcholine receptor titers of 5.70 nmol/L (normal < 0.21 nmol/L), supporting a diagnosis of myasthenia gravis in this patient. She was subsequently started on pyridostigmine.

Often, patients with thymoma experience paraneoplastic syndrome-related symptoms prior to thymectomy, and in many cases thymectomy is curative. However, in the case presented, we examine a patient that was asymptomatic prior to surgery and subsequently reported the onset of symptoms following what we suspect was an exacerbation due to general anesthesia and pain control medications. We argue that all patients with thymoma should undergo systematic evaluation and treatment of paraneoplastic syndromes, regardless of clinical symptoms and prior to surgery, in order to improve patient quality of life and hospital outcomes.

## Introduction

Thymomas are malignant cancers of the thymus, a primary lymphoid organ of the anterior mediastinum where T-lymphocytes mature immunologically [1]. It is known that the thymus naturally involutes with age and decreases in functionality while retaining some capacity to regenerate; however, there is no well-defined age at which it begins this process [1,2]. In some cases, the epithelium of the thymus undergoes abnormal cell proliferation and creates thymic epithelial neoplasms [3]. The thymoma is one such neoplasm and is made of both lymphocytes and epithelial cells, giving rise to multiple classes of thymoma depending on the varying degrees of each [4]. Although rare, with an incidence of around 1.5 cases per million, this cancer accounts for around 20% of malignancies in the adult mediastinum [3,4]. The prevalence of thymomas is comparable between males and females and the median age of diagnosis differs between races, with 48 years in Black patients and 58 in White patients [5,6]. The Masaoka Staging System allows for tumor staging, prognostic predictions, and treatment guidance of thymomas [7]. Treatment modalities available are surgical resection, systemic treatments, radiotherapy, and chemotherapy depending on each case [6]. Thymomas are often associated with other conditions such as myasthenia gravis (MG), pure red cell aplasia, immunodeficiency, and multi-organ autoimmunity [4,8].

Myasthenia gravis is a common paraneoplastic syndrome related to thymomas [8,9]. MG is an autoimmune disorder that presents with weakness on repetitive use of certain muscle groups, such as ocular, bulbar, proximal muscles of the extremities, and respiratory muscles to varying degrees and in varying combinations [9,10]. The variable, fatigable weakness present in MG is a manifestation of autoimmune IgG antibodies targeted against muscle nicotinic acetylcholine receptors (AChR) [9] disrupting postsynaptic neuromuscular transmission [10]. The incidence rate of MG as a paraneoplastic syndrome in thymoma is estimated at around 0.25-2 cases per million, with a trend of increasing prevalence suspected to be due to increased life expectancy as a result of effective treatment [10,11]. MG is diagnosed clinically by history and physical exam findings. Diagnosis can be aided by electrophysiological studies and serum autoantibody titers, most commonly the AChR antibodies. Treatment is chosen on a case-by-case basis according to each patient's profile and personal wishes [6]. Herein, we present a case of MG in a 36-year-old female with a previously resected thymoma who initially presented for iron deficiency anemia.

## Case presentation

Thymoma

In January 2022, the patient, a 36-year-old female with a significant past medical history of obesity, vitamin D deficiency, chronic left shoulder pain, intermittent asthma, and iron deficiency anemia acquired a coronavirus disease (COVID-19) infection, which led her to develop dyspnea and chest pain. She presented to the emergency department for worsening symptoms and underwent an extensive work-up, including a computed tomography (CT) scan of the chest. The patient was incidentally found to have a left upper lobe (LUL) lung mass measuring 2.8 cm x 4.4 cm x 3.6 cm that was directly bordering the pulmonary artery. On follow-up with pulmonology, a CT-guided biopsy and positron emission tomography (PET)-CT scan were ordered to evaluate the LUL lung mass.

**Figure 1:**
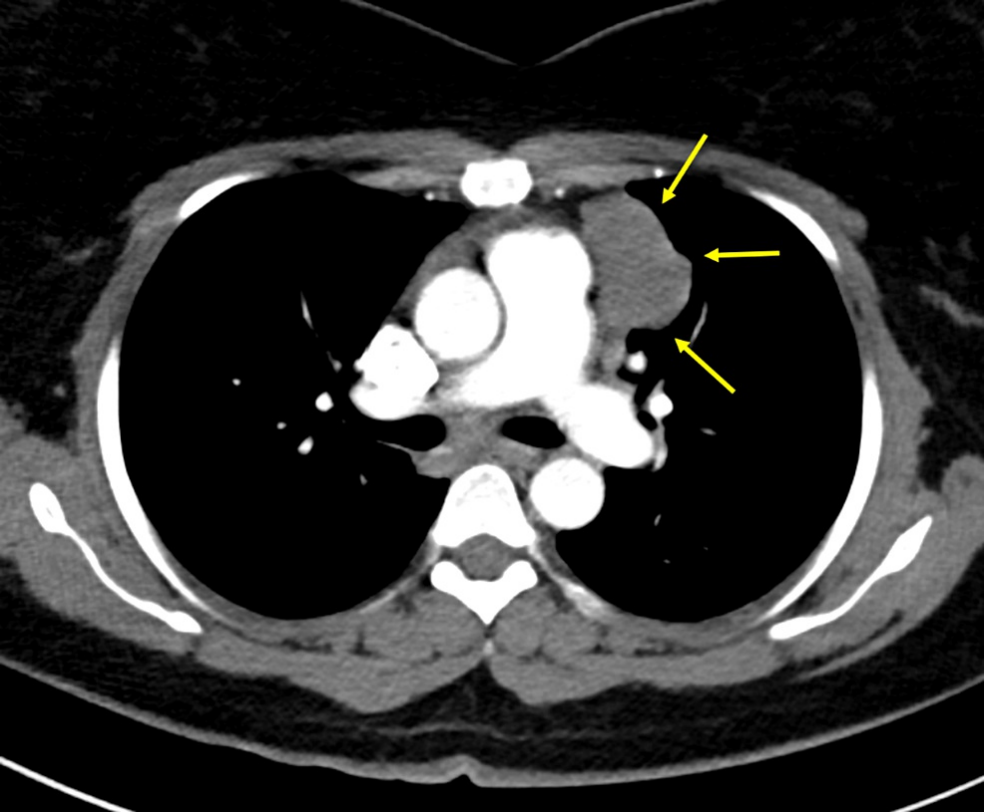
The lesion abuts the third left costal cartilage without invasion or erosion. It also, medially abuts the main pulmonary artery with a thin fat plane of separation.

**Figure 2:**
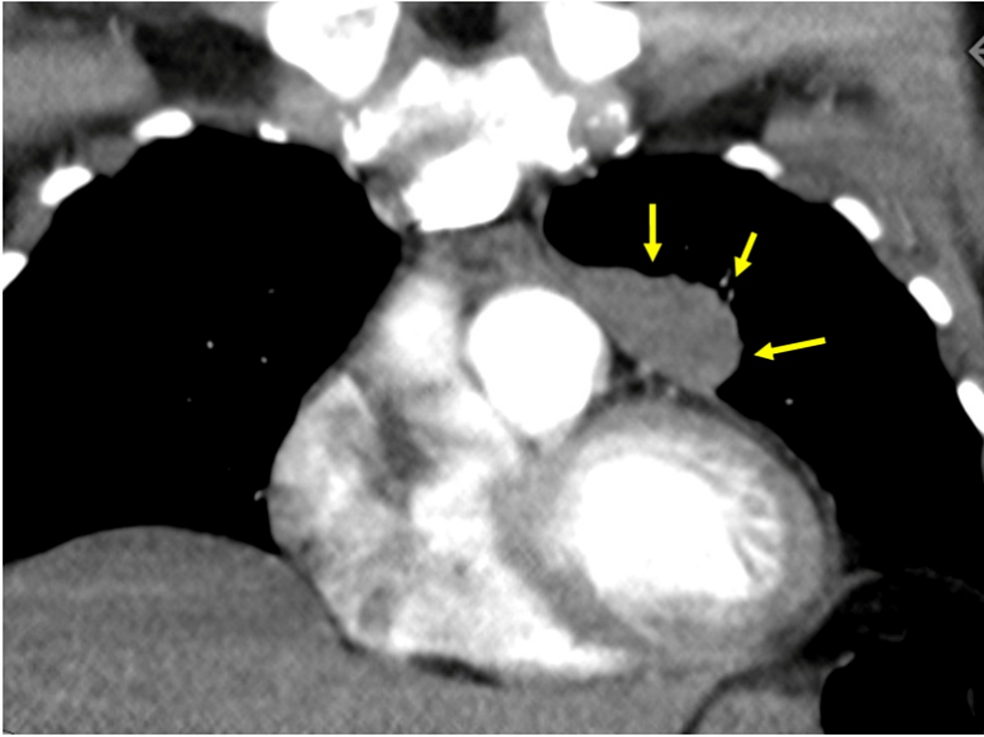
A second view of how the lesion abuts the third left costal cartilage without invasion or erosion, while also medially abutting the main pulmonary artery with a thin fat plane of separation.

**Figure 3:**
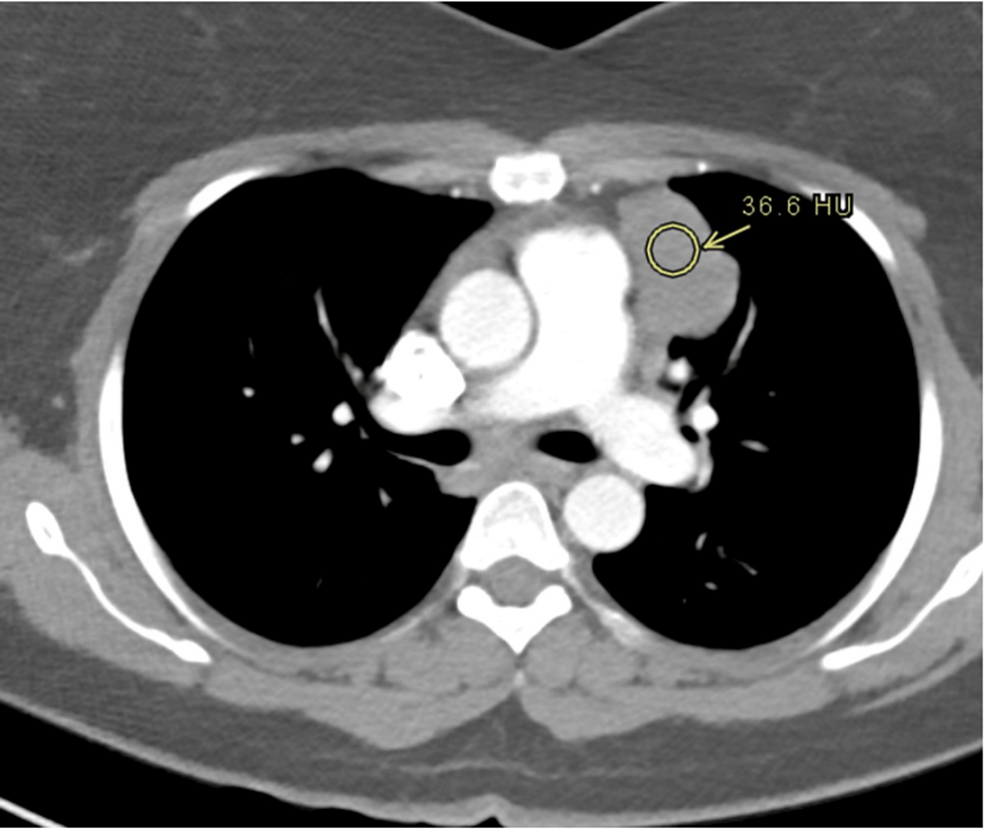
Well-defined solid left anterior mediastinal mass measuring 4.5 x 2.8 cm. There is no calcification, fat or cystic changes.

In February 2022, the patient underwent PET-CT scan imaging, which re-demonstrated a metabolically active LUL lung mass with a maximum standardized uptake value of 11.3, indicating potential cancerous involvement. She proceeded to undergo a CT-guided biopsy. Images from the CT-guided biopsy are supplied in Figures 1-3. On pathologic examination, the biopsied sample was found to have features most consistent with thymoma, with predominant lymphoid tissue. The biopsied sample had low cell yield and thus was not ideal for flow cytometry immunophenotypic testing. The patient was then referred to cardiothoracic surgery.

At the end of March 2022, the patient underwent left robotic thoracoscopy with a plan for complete thymectomy. Intra-operatively, the tumor was upstaged from a modified Masaoka Stage II to a Stage III thymoma due to visualized extension into the lung parenchyma. It was decided to proceed with extended resection of the thymoma *en bloc*, including complete removal of the LUL of the lung, and partial resection of the left phrenic nerve and pericardium. Complete resection was achieved, with negative surgical margins of 0.5 mm. Diagnosis of thymoma was confirmed on pathologic examination. The mass was found to demonstrate World Health Organization (WHO) B2 and B3 components and measured 5.2 cm in greatest dimension. One lymph node was dissected and was found to be negative for malignant disease. Following surgery, the patient was prescribed paracetamol and gabapentin for pain control.

In April 2022, a thoracic oncologist evaluated the patient and referred her to radiation oncology, as she was a candidate for radiotherapy according to the National Comprehensive Cancer Network (NCCN) guidelines [12]. The radiation oncologist recommended adjuvant radiotherapy for this patient and currently, the patient will begin said regimen in the near future.

Myasthenia gravis

In June 2022, the patient visited her hematologist for follow-up on iron-deficiency anemia. The patient reported that she was feeling fatigued and was experiencing weakness with repetitive motion on review of systems. Other symptoms present were headaches, dysphagia and blurred vision. The physician noted that the patient had horizontal nystagmus and appeared fatigued during the physical exam. MG was suspected and anti-acetylcholine receptor (anti-AChR) antibody titers were ordered. Titers were elevated with a result of 5.70 nmol/L with the institutional reference range being <0.21 nmol/L for normal results. Gabapentin prescribed to the patient following her surgery was discontinued and the patient was counseled about the effects of certain medication classes on MG, prescribed pyridostigmine, and was then referred to neurology.

In July 2022, the patient presented to the neurology clinic, where she reported experiencing vision loss, blurriness, facial drooping, difficulty swallowing, dyspnea, and fatigue for the past four months. The timeline of symptom appearance overlaps with the post-thymectomy period. Physical exam findings, including a comprehensive neurological exam, were normal with the exception of bilateral eyelid ptosis that worsens with sustained upward gaze. The patient initially did not improve after the prescription of pyridostigmine due to non-compliance, as reported by the patient. Pyridostigmine was continued by neurology with dose modification for better tolerance.

## Discussion

Thymoma and MG mechanisms

Thymomas and MG are quite interlinked: 50% of thymoma patients develop MG and 10%-20% of MG patients are found to have thymomas [13]. This interconnection is due to the link between the function of thymic tissue and the pathogenesis of myasthenia gravis. Immunological maturation takes place when the T cells interact with cell surface proteins on the thymic tissue cells in order to be able to differentiate between self and non-self-antigens [1]. In the case of thymoma-associated MG, it is hypothesized that thymomas hold epitopes that can cross-react with certain skeletal muscle proteins. These epitopes are the ryanodine receptor (RyR), titin, and acetylcholine receptor [14]. The most implicated antibody in the pathogenesis of MG is the anti-AChR antibody; however, the majority of paraneoplastic thymoma-associated MG cases also have anti-titin or anti-RyR antibodies [14].

Due to the nature of the autoantibodies present in MG, certain drugs could be detrimental to the patient’s health and could induce a myasthenic crisis [15]. These drugs include neuromuscular blockers, inhalation anesthetics, and gabapentin [15, 16]. We suspect that the use of gabapentin may have caused our patient to have an increase in MG symptoms since the time of the surgical resection when it was prescribed to her for pain control. The patient underwent surgical resection under general anesthesia, which may have also caused an exacerbation of symptoms. Here, we highlight the importance of awareness of MG as a common paraneoplastic syndrome in thymoma. Our purpose for presenting these findings is to optimize patient care and improve quality metrics.

Surgery and anesthesia recommendations

There are no formal guidelines or recommendations for patients undergoing surgery with general anesthesia that are known to have a thymoma but have an unknown myasthenia gravis status. Decisions regarding MG workup in patients with confirmed thymomas pre-thymectomy are largely institution-based and vary in purpose, with some being used to guide further management and others being used to monitor trends [17-19]. For patients with known MG undergoing surgery, the standard of care is to employ plasmapheresis or intravenous immunoglobulin (IVIG) infusions prior to surgery in order to extract complex pathogenic autoantibodies from the patient's sera [20]. This is done to keep the patient safe during general anesthesia and to protect them from post-thymectomy MG symptom exacerbation or crisis [20].

In our patient’s case, the titer status was unknown and, thus, she received no prophylactic pre-operative intervention against an exacerbation of symptoms or a crisis. Although MG is typically a clinical diagnosis, this case highlights the importance of maintaining a high index of suspicion in patients with thymoma with or without clinical manifestations prior to undergoing surgery. In many cases, surgical resection greatly reduces MG symptoms or MG may go into remission. However, we argue that patients should be monitored following surgery due to the potential for exacerbation of MG.

Quality improvement and safety

The purpose of presenting this case is to emphasize the need for better safety and quality improvement practices. The lack of guidelines or recommendations for pre-operative workup and peri-operative management of MG in patients with thymomas could lead to deleterious effects, especially given the context of general anesthesia and the stress of surgery. We believe that it would be beneficial to have a guideline in place that mandates the testing of common MG-associated antibodies prior to thymectomy, and as part of pre-operative management in the case of a patient with thymoma. Antibody testing is generally time- and cost-efficient. Additionally, it is important to obtain a neurology consult for a comprehensive clinical exam and confirmation of clinical signs. We believe systematic evaluation of paraneoplastic MG in patients with thymoma would improve patient safety and allow for optimal medical management.

## Conclusions

Myasthenia gravis as a paraneoplastic disease in patients with thymoma requires careful attention. Identifying these patients prior to undergoing surgery and general anesthesia is paramount in ensuring proper management and care. In this case, we highlight the importance of screening for MG in a thymoma patient before thymectomy and the need for guidelines to be set in place. Avoiding myasthenic crises and complications helps in ensuring a better quality of life for patients and in ensuring satisfactory health outcomes. Further cross-team collaboration in this area is important in the care of such patients, and for health, institutions to establish guidelines that guarantee no patient falls in between the cracks of missed conditions.
